# Point-of-care ultrasound competency of doctors working in Cape Town emergency departments

**DOI:** 10.4102/safp.v67i1.6151

**Published:** 2025-07-16

**Authors:** Karen Ferreira, Clint Hendrikse, Heinri Zaayman, Elaine Erasmus, Daniël J. van Hoving

**Affiliations:** 1 Department of Family, Community and Emergency Care, Faculty of Health Sciences, University of Cape Town, Cape Town, South Africa; 2 Department of Emergency Medicine, Faculty of Health Sciences, Stellenbosch University, Cape Town, South Africa

**Keywords:** ultrasound, training, competence, credentialling, South Africa, emergency medicine

## Abstract

**Background:**

Point-of-care ultrasound (PoCUS) is a core competency in emergency medicine, and its use in other primary healthcare settings is growing. The study investigates the PoCUS competency, training and qualifications of doctors working in public emergency departments.

**Methods:**

An online survey was distributed to doctors at five public Cape Town emergency departments, followed by a practical assessment of an Extended Focused Assessment with Sonography in Trauma (eFAST) and a basic cardiac ultrasound examination. Descriptive and inferential statistics are presented.

**Results:**

All participants had attended an in-person PoCUS course before, and 45 (83.3%) were trained by supervisors at work. Eleven participants (20.4%) were credentialled. In the practical assessment, 73.5% were rated as competent in eFAST and 55.9% in basic cardiac ultrasound. The median scores were 80.4% (eFAST) and 76.9% (cardiac ultrasound). Credentialled participants were more likely to achieve a pass mark (> 60%) in eFAST (*p* < 0.001) and cardiac ultrasound (*p* < 0.001).

**Conclusion:**

All the emergency department doctors who use PoCUS had received formal PoCUS training, and the majority of PoCUS providers had an adequate skill level in the applications tested. The credentialled providers performed better overall. There is a need for further research to investigate the persistently low credentialling rate and potential solutions, not only among practitioners in emergency departments but also generalists and primary care practitioners.

**Contribution:**

Our study provides a unique snapshot of the PoCUS skills of junior doctors and trainees in public Cape Town emergency departments.

## Introduction

Point-of-care ultrasound (PoCUS) is a well-established modality in the emergency medicine community. The African Federation for Emergency Medicine (AFEM) has recommended that PoCUS be incorporated into all African emergency care programmes for health practitioners.^1^ It is now considered standard of care and is a core competency in the practice of emergency medicine, and its use in other primary healthcare settings is growing.^2,3^ Point-of-care ultrasound is a relatively cost-effective, safe and easy-to-use modality. It lacks ionising radiation and can have a positive impact on diagnostic accuracy, clinical decision-making and length of stay in the emergency department (ED).^4,5,6,7,8,9^ Point-of-care ultrasound has an increasingly broad scope of applications, including diagnostic, procedural and monitoring. It can also be integrated into resuscitation and other therapeutic applications.^10,11^ The utilisation of PoCUS in resource-limited settings can have a significant impact on healthcare delivery and equity. In settings without traditional radiologic and laboratory capabilities, PoCUS can aid in diagnostics, patient management decisions and timely treatment of potentially life-threatening conditions.^12,13,14,15,16,17,18,19^ In a low- and middle-income country (LMIC) setting, where a large proportion of healthcare is delivered by primary healthcare practitioners, there is an increasing need to adopt PoCUS in clinical practice.^12^

There is currently no global standardised PoCUS curriculum or training standards.^20^ The Emergency Medicine Society of South Africa (EMSSA) established a programme for PoCUS training and credentialling in South Africa in 2008. The Emergency Medicine Society of South Africa follows the international approach to PoCUS training, which comprises four stages: introduction, gaining experience, confirmation of competency, and skills maintenance.^20,21^ It is almost universally accepted that PoCUS providers need to undergo a credentialling process to prove their competency in the theoretical and practical applications of PoCUS before using the modality independently.^22^ The Emergency Medicine Society of South Africa defines competency in PoCUS as:

[*K*]nowing when to use a specific ePoCUS application; successful ability to acquire and optimise ultrasound images; and correct interpretation of the ePoCUS exam findings along with the successful integration of these into individual patient care plans.^21^

In South Africa, credentialling involves attending a basic PoCUS course, followed by reflective practice and finally a written and practical exam. Once candidates pass the exam, they are accredited to use basic PoCUS independently in the clinical setting.^21^ Inappropriate or incorrect use of PoCUS can cause patient harm, unnecessary investigations and increased medico-legal liability.^23^ However, there is limited evidence that credentialling is an accurate predictor of safe PoCUS use in day-to-day practice.^21^

Despite the emphasis on PoCUS providers achieving competency, there has been a poor uptake of credentialling in both LMICs and high-income countries (HICs).^23,24,25^ A number of studies showed credentialling rates ranging from 19.7% to 43.9% among emergency medicine trainees and specialists.^21,22,23^ Of note is that high numbers of non-credentialled providers continue to use PoCUS independently for diagnosis and clinical decision-making.^24,25,26^

Much of the available literature on PoCUS training and credentialling focuses on emergency medicine trainees (residents or registrars) and specialists.^7,25,26^ In the South African setting, EDs are staffed by a wide range of providers, with a large part of the workforce consisting of junior doctors and medical officers.^27^ Although EMSSA recommends that junior doctors should receive formal PoCUS training, it acknowledges that most core PoCUS competencies are frequently taught and practised in the work environment and states that it is essential to have a supervised PoCUS education programme in place for junior doctors in the ED.^21^ Not much is known about the current use of PoCUS by doctors working in South African EDs, including their level of training and credentialling, frequency of use and accuracy. The aim of the study was to investigate the PoCUS competency, training and qualifications of doctors working in EDs who use PoCUS. A secondary aim was to compare the PoCUS competency of ED doctors who have completed any credentialling versus those who have not.

## Research methods and design

### Study design

We conducted a multi-centre cross-sectional study.

### Setting

Our study setting was district hospitals in Cape Town, South Africa. The Cape Town metropolitan district serves a population of over 4 million people with a population density of 1641 people per square kilometre.^28^ Primary level health services are provided through local clinics and 24-h community health centres. Higher-level services are generally provided at hospitals that are categorised as: district (level 1), regional (level 2) or tertiary and/or central (level 3) hospitals.^29^

### Study population and sampling strategy

The study population consisted of doctors (including community service medical officers, medical officers and emergency medicine registrars) working in public EDs in Cape Town who use PoCUS. The approximate size of our study population was 100 people. This approximation is based on the number of community service medical officers, permanent and regular locum medical officers and emergency medicine registrars who were working at each facility at the time of data collection.

A formal sample size calculation was not carried out as a purely descriptive study was foreseen.

Convenience sampling was used. One regional and four district hospitals in Cape Town were sampled, where emergency physicians are employed and where doctors have access to accredited PoCUS trainers (physicians who are accredited in basic PoCUS and have qualified as PoCUS instructors). The five facilities were Khayelitsha Hospital, Mitchells Plain Hospital, Victoria Hospital, New Somerset Hospital (regional level) and Karl Bremer Hospital. These public EDs manage undifferentiated patients (medical and trauma) of all ages.

### Data collection

The study was performed from April to June 2023 and included an online survey and a practical assessment. The REDCap electronic data capture tool^30,31^ was used for the online survey, which included online consent, demographics, PoCUS applications used and their frequency, PoCUS training level and credentialling status. The survey instrument (Online Appendix 1) was piloted on non-participant ED doctors and modified as required before starting data collection.

The survey link was distributed to the emergency medicine consultants at each facility via WhatsApp. Consultants then shared the link on their departmental WhatsApp groups with weekly reminders over a four week period. Participation was voluntary and consultants did not know who had or had not completed the survey. Only the primary author had access to the survey responses. Following completion of the survey, participants who had indicated interest in participating in the practical session were contacted via email and invited to attend a practical assessment. One or two practical sessions were held at each facility, depending on the number of participants. The format of the practical assessment was based on the EMSSA credentialling examination format. Each participant attended a 30-min individual session with two examiners. A total of four examiners participated in the study, all senior accredited EMSSA PoCUS examiners. Examiners were blinded to the participants’ level of training and credentialling and were not employed at the same facility as the participants. Participants were given two clinical scenarios and asked to perform the Extended Focused Assessment with Sonography in Trauma (eFAST) examination and a basic cardiac ultrasound examination within the 30-min timeframe. These two modalities were chosen because they were the most commonly used core PoCUS applications, based on anecdotal experience as well as a previous survey carried out among doctors working in African EDs.^23^ Participants were also shown two ultrasound clips pertaining to the scenario, which demonstrated a pneumothorax (presence of a lung point) and a dilated cardiomyopathy. The marking sheets were created by the examiners and modelled on the EMSSA credentialling exam marking sheet (Online Appendix 2 and 3). Ultrasound machines from the relevant facilities were used to ensure familiarity. Models were male volunteers from the relevant facility and 3 of the 10 models had pathological findings (pleural effusion and/or ascites). Participants were given a percentage score (calculated by tallying their marks), with a pass mark being 60% or more (the same as EMSSA criteria). The examiners decided on the marks through consensus.

### Data analysis

Descriptive statistics were used to describe the participants’ demographics, level of training and credentialling, frequency of PoCUS use, and the results of the practical assessment. Our data did not follow a normal distribution; therefore, non-parametric tests were used. The Mann–Whitney *U* test was used to compare the percentage scores between groups (credentialled and non-credentialled). The percentage scores were dichotomised into pass and fail categories (60% benchmark) before comparing the pass and fail categories between groups with McNemar’s test. Finally, the percentage scores were compared to the participants’ frequency of use of eFAST and cardiac ultrasound (as per the survey), using the Mann–Whitney *U* test and Kruskal–Wallis test respectively. Data analysis was performed using SPSS Statistics for Windows, Version 28.0 (IBM Corp. Released 2021. Armonk, NY: IBM Corp.).

### Ethical considerations

An application for full ethical approval was made to the University of Cape Town Human Research Ethics Committee and ethics consent was received on 16 March 2023. The ethics approval number is HREC REF 160/2023.

## Results

**FIGURE 1:**
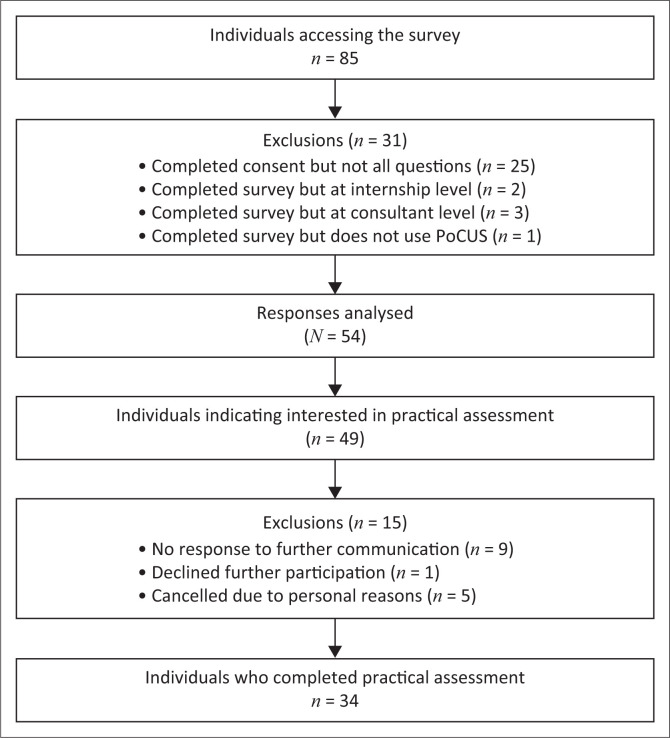
Flow diagram of study participants.

Eighty-five respondents accessed the online survey. Fifty-four survey responses (~ 54% of the study population) were analysed (Figure 1). The mean age of participants was 30.7 years (standard deviation ± 5.4). There was a predominance of female participants (*n* = 31, 57.4%), while 15 (27.8%) were community service medical officers, 29 (53.7%) medical officers, and 10 (18.5%) emergency medicine registrars.

All participants (*n* = 54, 100.0%) had previously attended an in-person PoCUS course (EMSSA and/or non-EMSSA), and most participants (*n* = 45; 83.3%) received training from their supervisors at work (Table 1). Forty-three participants (79.6%) were not credentialled, and 11 (20.4%) had passed the EMSSA credentialling examination.

**TABLE 1:** Training experience of survey respondents.

PoCUS training	All participants (*N* = 54)†		Community service medical officers (*n* = 15)†		Medical officers (*n* = 29)†		Registrars (*n* = 10)†	
	*n*	%	*n*	%	*n*	%	*n*	%
Any in-person PoCUS course	54	100.0	15	100.0	29	100.0	10	100.0
Taught by supervisors at work	45	83.3	14	93.3	27	93.1	5	50.0
EMSSA Core e-PoCUS course	37	68.5	6	40.0	22	75.9	9	90.0
Online resources and tutorials	37	68.5	6	40.0	26	89.7	5	50.0
Non-EMSSA in-person PoCUS course	14	25.9	2	13.3	10	34.5	2	20.0
Part of undergraduate training	2	3.7	1	6.7	1	3.4	0	0.0
EMSSA Advanced PoCUS course	1	1.9	0	0.0	1	3.4	0	0.0
Other‡	4	7.4	0	0.0	3	10.3	1	10.0

PoCUS, point-of-care ultrasound; EMSSA, Emergency Medicine Society of South Africa; e-PoCUS, emergency point-of-care ultrasound.

† , Participants could select more than one option;

‡ , Includes echocardiography course, focus assessed transthoracic echocardiography course, unspecified PoCUS symposium.

The most frequently used PoCUS applications were assessment for deep venous thrombosis (*n* = 54; 100%), eFAST (*n* = 53; 98.1%), basic cardiac ultrasound (*n* = 52; 96.3%), and lung ultrasound (*n* = 52; 96.3%) (Table 2).

**TABLE 2:** Frequency of use of point-of-care ultrasound applications with core point-of-care ultrasound modules in grey (*N* = 54).

Application	Used		Frequency of use									
			Daily		Weekly		Monthly		Less than monthly		Never	
	*n*	%	*n*	%	*n*	%	*n*	%	*n*	%	*n*	%
Deep venous thrombosis	54	100.0	4	7.4	35	64.8	11	20.4	4	7.4	0	0.0
eFAST	53	98.1	24	44.4	23	42.6	3	5.6	3	5.6	1	1.9
Cardiac including inferior vena cava assessment	52	96.3	18	33.3	26	48.1	6	11.1	2	3.7	2	3.7
Lung	52	96.3	18	33.3	30	55.6	3	5.6	1	1.9	2	3.7
Central intravenous access	49	90.7	1	1.9	11	20.4	22	40.7	15	27.8	5	9.3
Abdominal aorta aneurysm assessment	48	88.9	3	5.6	21	38.9	18	33.3	6	11.1	6	11.1
Obstetric and gynaecological	47	87.0	10	18.5	18	33.3	9	16.7	10	18.5	7	13.0
Peripheral intravenous access	47	87.0	3	5.6	8	14.8	20	37.0	16	29.6	7	13.0
Other procedural†	46	85.2	1	1.9	17	31.5	22	40.7	6	11.1	8	14.8
Genito-urinary including testicular	37	68.5	0	0.0	7	13.0	12	22.2	18	33.3	17	31.5
FASH	36	66.7	3	5.6	13	24.1	11	20.4	9	16.7	18	33.3
Hepatobiliary	36	66.7	2	3.7	13	24.1	14	25.9	7	13.0	18	33.3
Ocular	33	61.1	0	0.0	9	16.7	8	14.8	16	29.6	21	38.9
Regional anaesthesia	23	42.6	1	1.9	1	1.9	6	11.1	15	27.8	31	57.4
Gastro-intestinal tract	19	35.2	0	0.0	4	7.4	6	11.1	9	16.7	35	64.8
Musculoskeletal and soft tissue	2	3.7	0	0.0	2	3.7	0	0.0	0	0.0	52	96.3

eFAST, extended focused assessment with sonography in trauma; FASH, focused assessment with sonography for HIV-associated tuberculosis.

† , Includes all procedures other than vascular access.

A total of 34 participants completed the practical assessment (Figure 1). The median score in the eFAST assessment was 80.4%, with an interquartile range (IQR) of 23.6%. Thirty-one participants (91.2%) achieved a pass mark (score ≥ 60%), which included all of the credentialled participants (11/11) and 87% (20/23) of non-credentialled participants.

The median score in the basic cardiac ultrasound assessment was 76.9% (IQR 29.6%). Twenty-six participants (76.5%) achieved a pass mark; which included all of the credentialled participants (11/11) and 65.2% (15/23) of non-credentialled participants (Table 3). All registrars (10/10) achieved a pass mark in both the eFAST and basic cardiac ultrasound assessments (Table 4).

**TABLE 3:** Results of practical assessments.

Result All *n* (%) unless otherwise indicated	All participants (*n* = 34)	Credentialled participants (*n* = 11)	Non-credentialled participants (*n* = 23)	*p*-value
**eFAST**				
Pass (≥ 60%)	31 (91.2)	11 (100.0)	20 (87.0)	< 0.001
Median (IQR) score (%)	80.4 (23.6)	84.6 (20.0)	80.0 (29.5)	0.188
**Cardiac**				
Pass (> 60%)	26 (76.5)	11 (100.0)	15 (65.2)	< 0.001
Median (IQR) score (%)	76.9 (29.6)	90.2 (13.8)	74.0 (21.9)	< 0.001

eFAST, extended focused assessment with sonography in trauma; IQR, interquartile range.

**TABLE 4:** Results of practical assessments according to the category of practitioner.

Result All *n* (%) unless otherwise indicated	Community service medical officers (*n* = 5)		Medical officers (*n* = 19)		Registrars (*n* = 10)	
	*n*	%	*n*	%	*n*	%
eFAST pass (≥ 60%)	4	80.0	17	89.5	10	100.0
Cardiac pass (> 60%)	2	40.0	14	73.7	10	100.0

eFAST, extended focused assessment with sonography in trauma.

The associations between percentage scores in the assessments and frequency of PoCUS use reported in the survey were not significant (eFAST *p* 0.837; Cardiac ultrasound *p* 0.158).

**FIGURE 2:**
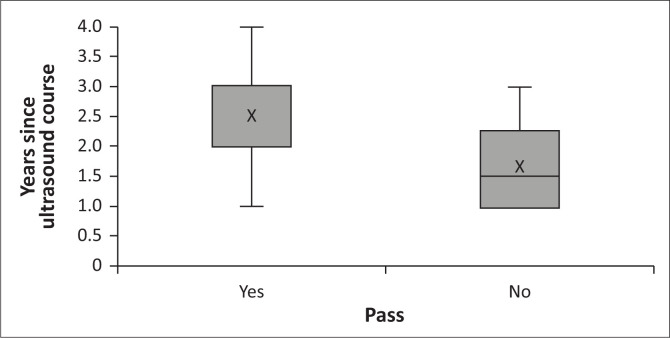
Number of years since the most recent ultrasound course was completed, divided according to the outcome of practical assessment (pass mark ≥ 60%).

Participants’ results in the practical assessment were also plotted against the number of years since the participant’s most recent ultrasound course was completed (Figure 2).

## Discussion

We set out to investigate the PoCUS training and competency of public Cape Town ED doctors and found that credentialled PoCUS providers performed better than the non-credentialled group. Although all participants had received formal PoCUS training before, only 20% of participants had passed a PoCUS credentialling exam. Participants performed better in the eFAST assessment than in the basic cardiac ultrasound assessment.

All survey respondents (100%) had attended an in-person PoCUS course prior to the study, with 83% indicating that they also received training from their supervisors at work. This is higher than reported rates of PoCUS training in Africa, where a 2022 survey indicated that 78% of African emergency doctors received formal hands-on training.^23^ It is also higher than in HICs, where 73% (South Korea) to 86% (Australia and the USA) of emergency medicine specialist trainees and specialists had received some formal PoCUS training.^26,32,33^ This could be because our study population consisted only of junior doctors and emergency medicine trainees, most of whom started practising in the PoCUS era, compared to studies that included qualified emergency physicians who might have completed their training before PoCUS use became widespread. It is encouraging that such a high proportion of PoCUS users have had formal PoCUS training, but despite this, the uptake of credentialling remains poor.

The low credentialling rate (20%) among respondents is a concern. This aligns with a 2018 study which also demonstrated a credentialling rate of 20% among Cape Town doctors.^24^ Our credentialling rate was substantially lower than the 44% reported among African emergency doctors, but was similar to rates of 15% to 18% reported in two Australasian studies.^23,25,26^ Some HIC studies do report very high credentialling rates (82.4% and 100% respectively in two USA studies), but these were only achieved after the implementation of study interventions (namely, intensive training and credentialling programmes), highlighting the fact that poor credentialling uptake is a global problem and not limited to the African setting.^34,35^

It is concerning that the credentialling rate has not increased since the 2018 study.^24^ Time constraints and access to supervisors and machines were then the predominant barriers to credentialling.^24^ In 2021, EMSSA changed the requirements for credentialling and removed the compulsory logbook with a set number of proctored ultrasounds.^21^ This should have mitigated the impact of time constraints and the access barrier to supervisors but has not translated into increased uptake of credentialling. It is possible that with the changes in credentialling requirements and examination format, the barriers no longer lie in qualifying for the credentialling exam but rather in passing the exam. EMSSA’s credentialling pass rate was 51% in 2021 and 53% in 2022 (personal communication: Western Cape and Northern Cape representative of EMSSA ultrasound special interest group). This may be a result of inadequate preparation, lack of familiarity with the exam format or a higher standard of exam than expected.

The most frequently used PoCUS applications correspond with the EMSSA curriculum’s core PoCUS modules (eFAST, basic cardiac ultrasound, lung ultrasound, abdominal aorta assessment, limited deep venous thrombosis assessment and ultrasound-guided venous access).^21^ Although all participants reported using DVT assessment, most used eFAST, basic cardiac ultrasound and lung ultrasound far more frequently. Our study population comprised a relatively junior cohort of doctors, and therefore, the more frequent use of core PoCUS applications (versus advanced applications) is logical.

The majority of participants were considered competent in the use of eFAST (91%) and in basic cardiac ultrasound (77%). It is not surprising that participants performed better in eFAST, as basic cardiac ultrasound is technically more difficult. In a recent study among Finnish emergency physicians, participants considered eFAST one of the easiest PoCUS applications to gain competency in, while focused cardiac ultrasound was the most difficult modality to become competent in.^36^

The credentialled group performed better than the non-credentialled group in both assessments. This is likely because the credentialled group would have carried out more focused learning and practice in preparation for their credentialling exam. Additionally, in some EDs, only credentialled doctors are allowed to use PoCUS without supervision, which may give credentialled doctors more opportunities to practise PoCUS than their non-credentialled colleagues (personal communication: Associate Professor Clint Hendrikse, Division of Emergency Medicine at the University of Cape Town).

When comparing the number of years since a participant had completed their most recent ultrasound course versus their performance in the practical assessment, we found a trend towards lower scores and greater variability in performance among those who had completed their ultrasound course most recently. This underlines the need for longer-term reflective practice following initial PoCUS training before an individual can be considered competent in PoCUS.

There are limited studies that assess the skill level of PoCUS providers using a similar methodology to ours, making it difficult to compare results. Long et al. conducted a similar study in the US, where providers’ PoCUS skills (in eFAST, cardiac, aorta and early pregnancy ultrasound) were assessed in an examination setting. They reported a pass rate of 71%, although this was among qualified emergency physicians rather than trainees.^32^ Multiple other studies have looked at the accuracy of images obtained by providers in a clinical setting, but most of these studies involved an education or training intervention followed by measurements of accuracy and other outcomes.^7,37,38^ Our study therefore provides a unique snapshot of the PoCUS skills of junior doctors and trainees.

## Limitations

Our study’s results are limited for various reasons. Our pragmatic sampling strategy and survey response rate resulted in a small sample size. The small sample size and low number of credentialled participants resulted in an underpowered study, and even though statistically significant results were demonstrated, the risk of Type II errors should not be overlooked. Selection bias could have been introduced by including only emergency medicine physician-led EDs. We chose to include these sites because the clinicians at these EDs were more likely to have received PoCUS training and use PoCUS regularly. Similarly, the inclusion of only facilities in the Western Cape may have introduced further selection bias and limited the generalisability of our results to other settings. The blinding of examiners to participants’ credentialling status was not optimal and could also have introduced bias. Although there was no disclosure of participants’ credentialling status before or during the assessments, our examiners had encountered many of the participants previously in PoCUS courses, clinical settings or clinical examinations. The marking sheet was intended to facilitate objective assessments and mitigate this potential bias. Different ultrasound machines and models were used for the practical assessments at the different sites, making the assessment less standardised across different sites. The potential effect thereof was mitigated by using standardised clinical scenarios, clips and marking sheets. The examiners also ensured that candidates knew how to use the machine before they started their assessment and assisted the candidates with changing settings on the machine when required. In addition, using a single examination to assess a practitioner’s competency in PoCUS may not be sufficient, but our study mimics the current credentialling format. Finally, participants understandably experienced some anxiety and apprehension during the practical assessment, which they likely do not experience when using PoCUS as part of their routine clinical work. This could have decreased their level of performance and should be considered when interpreting the results. Despite these limitations, we feel that the study results still allow us to gain a reasonable idea of the PoCUS competency of doctors working in public EDs in Cape Town.

## Recommendations

Our study indicated that junior doctors and medical officers working in public EDs had a good foundation of PoCUS knowledge and skills, with opportunities for further development.

We recommend that further research investigate the persistently low credentialling rate and potential solutions, not only among practitioners in EDs but also among generalists and primary care practitioners.

In addition, we suggest that South African medical schools and facilities training junior doctors (interns and community service medical officers) work towards implementing a PoCUS curriculum at the grassroots level (including undergraduate students) in order to equip new trainees with robust PoCUS skills in a healthcare landscape that will increasingly demand them.

Finally, we recommend that emergency medicine and primary care stakeholders implement strategies to collaborate and share PoCUS knowledge and skills, as opposed to our traditional siloed approach to healthcare. This could be greatly beneficial for clinicians, communities and our healthcare system.^39^ Clinicians working in primary care settings other than EDs could be empowered to use PoCUS for diagnostic and therapeutic purposes, allowing the community easier and more equitable access to care. This could also relieve some of the burden on regional and tertiary facilities through decentralising certain aspects of care.

## Conclusion

In our study, the majority of doctors working in public EDs who use PoCUS had received formal PoCUS training and had an adequate skill level in the applications we tested. Credentialled providers performed better. There is a need for further research to investigate the persistently low credentialling rate and potential solutions, not only among practitioners in EDs but also among generalists and primary care practitioners. Finally, emergency medicine and primary care practitioners need to collaborate and share PoCUS knowledge and skills.

## Dissemination of results

Results from this study were shared with participants and management teams at the data collection sites through a written report.
